# Menstrual characteristics and associations with sociodemographic factors and self-rated health in Spain: a cross-sectional study

**DOI:** 10.1186/s12905-023-02840-z

**Published:** 2024-02-03

**Authors:** Laura Medina-Perucha, Tomàs López-Jiménez, Georgina Pujolar-Díaz, Cristina Martínez-Bueno, Jordina Munrós-Feliu, Carme Valls-Llobet, Constanza Jacques-Aviñó, Anna Sofie Holst, Diana Pinzón-Sanabria, María Mercedes Vicente-Hernández, Andrea García-Egea, Anna Berenguera

**Affiliations:** 1 Fundació Institut Universitari per a la recerca a l’Atenció Primària de Salut Jordi Gol i Gurina (IDIAPJGol), Barcelona, Spain; 2 Universitat Autonoma de Barcelona, Cerdanyola del Vallès, Bellaterra, Spain; 3 Servei d’Atenció a la Salut Sexual i Reproductiva (ASSIR). Direcció Assistencial d’Atenció Primària, Institut Català de la Salut, Barcelona, Spain; 4 Sexual and Reproductive Health Care Research Group (GRASSIR), Barcelona, Spain; 5 Universitat de Barcelona, Barcelona, Spain; 6 Atenció a la Salut Sexual i Reproductiva (ASSIR) Muntanya/La Mina, Institut Català de la Salut, Barcelona, Spain; 7 Centro de Análisis y Programas Sanitarios (CAPS), Barcelona, Spain; 8 SomiArte Taller, Barcelona, Spain; 9 Departament d’Infermeria, Universitat de Girona, Girona, Spain

**Keywords:** Menstruation, Menstrual inequity, Menstrual health, Social inequities of health, Self-rated health, Women’s health, Spain

## Abstract

**Background:**

Evidence on how menstrual characteristics may differ based on socioeconomic factors and self-rated health is significantly scarce. The main aim of this study was to investigate the associations between menstrual characteristics, sociodemographic factors and self-rated health among women and people who menstruate (PWM) aged 18–55 in Spain.

**Methods:**

This cross-sectional study includes data from an online survey collected in March–July 2021 across Spain. Descriptive statistical analyses and multivariate logistic regression models were performed.

**Results:**

The analyses included a total of 19,358 women and PWM. Mean age at menarche was 12.4 (SD = 1.5). While 20.3% of our participants experienced a menstrual abundance over 80 ml, 64.1% reported having menstrual blood clots; 6.4% menstruated for longer than 7 days. 17.0% had menstrual cycles that were shorter than 21 days or longer than 35 days. Reports of moderate (46.3%) and high (22.7%) intensity menstrual pain were common. 68.2% of our participants experienced premenstrual symptoms in all or most cycles. The odds for lighter menstrual flow, shorter bleeding days and menstrual cycles were higher as age increased, and amongst participants with less educational attainment. Caregivers presented higher odds for abundant menstrual flow and longer menstruations. Reporting financial constraints and a poorer self-rated health were risk factors for abundant menstrual flow, menstrual blood clots, shorter/longer menstruations and menstrual cycles, premenstrual symptoms, moderate and intense menstrual pain.

**Conclusions:**

This study suggests that age, educational attainment, caregiving, experiencing financial hardship and a poorer self-rated health may shape or mediate menstrual characteristics. It thus highlights the need to investigate and address social inequities of health in menstrual research.

**Supplementary Information:**

The online version contains supplementary material available at 10.1186/s12905-023-02840-z.

## Background

The last few years have been crucial to draw attention towards the need to consider menstruation and the menstrual cycle as vital signs for the health of women and people who menstruate (i.e., gender non-confirming menstruators) (PWM). Menstrual health has been recently defined in an attempt to approach and conceptualize menstrual health in a holistic manner, as it also considers the access to accurate menstrual education, menstrual products and menstrual management facilities and services, a timely diagnosis for menstrual-related health conditions, having stigma and discrimination-free menstrual experiences, and being able to decide whether to participate in civil, cultural, economic, social and political spheres throughout the menstrual cycle [1]. Menstrual health is closely related to menstrual inequity, which refers to the systematic and avoidable differences in menstrual access and experiences, based on the intersection of social inequities of health amongst individuals and communities [2, 3].

The relationship between social and economic inequities and negative health outcomes [4, 5] and the feminization of poverty [6] is well-established. A growing literature, especially from Global South contexts but increasingly from the Global North, highlights that menstrual equity and health are especially compromised among socioeconomically vulnerable women and PWM, such as those living in situations of financial hardship [2, 7, 8], homelessness [9], displacement [10] and migration [11]. Socioeconomic inequities and a limited and inadequate access to healthy menstrual management can have a profound impact on reproductive [12], emotional [13, 14] and general [15] health outcomes. It is thus imperative to conduct research on menstrual health that considers social inequities of health and, particularly, socioeconomic factors. Besides, little is known about menstrual health patterns among women and PWM, as most research in Spain has focused on investigating menstrual disorders and specific populations [16, 17].

Menstrual characteristics encompass age at menarche, menstrual bleeding duration, menstrual cycle duration, menstrual bleeding abundance, premenstrual symptoms, menstrual pain, and menstrual blood clots. Previous evidence has demonstrated linkages between sociodemographic variables and menstrual characteristics [18, 19]. The decrease in age of menarche has been attributed to population changes in nutrition, physical activity and body fat [20–22], the exposure to endocrine disruptor chemicals [23–25], climate change [26], psychosocial stressors [27, 28], socioeconomic factors (e.g., family composition and income or place of residence) [20, 21, 28–30], factors related to race/ethnicity [22], among other factors [31–34]. Similarly, higher age, lower education level [19], as well as living in deprived areas are linked with experiences of heavy menstrual bleeding [35]. Menstrual cycle duration variates depending on age, ethnicity, and body weight [36]. Many sociodemographic characteristics have been reported related to menstrual pain, such as age [37, 38], body mass index [37, 39], low socioeconomic status [37, 38, 40], and family history of dysmenorrhea [41]. Premenstrual symptoms were more common in women with higher educational status [42] and experiencing stress [43]. Menstrual blood clots (those greater than 1 in. = 2,5 cm) are used as indicators of heavy menstrual bleeding [18, 44, 45] as well as a sign of adenomyosis [46].

On the other hand, self-rated health is a known proxy for health status [47, 48] and an indicator for health equity [48–51]. Considering that self-rated health is mediated by social, cultural, economic and political factors, it is necessary to contextualize the understanding of how self-rated health may be associated with health outcomes. In the area of menstrual health and equity research, Teperi and Rimpelä already suggested in 1989 that poor self-rated health was a determinant of menstrual pain in Finland [52]. However, to the authors’ knowledge, menstrual health research has not yet further explored the potential association between menstrual characteristics and self-rated health.

Having identified this gap the current article explores the intersection between menstrual characteristics, sociodemographic factors, and self-rated health. Understanding menstrual characteristics in context is necessary to menstrual health and equity research, particularly to highlight the needs of most vulnerable populations. This is particularly relevant at a time when menstrual policymaking is rapidly increasing and, often, failing to implement evidence-based policies. In order not to become tokenistic, policies should consider and respond to the needs of different groups of women and PWM. The main aim of this study was to investigate the associations between menstrual characteristics, sociodemographic factors and self-rated health among women and PWM aged 18–55 in Spain.

## Materials and methods

This is a cross-sectional study, part of the “Equity and Menstrual Health in Spain” project. This study adopts a critical and feminist perspective to public health research, and critically questions androcentrism and systemic sociopolitical inequities of health that impact women and PWM [53, 54].

### Setting

An online questionnaire was devised by the research team, given the lack of standardized measures available in our context. The team consists of interdisciplinary experts including psychologists, medical doctors, public health professionals, midwives. The questionnaire was developed during several meetings and it was piloted before data collection. Data were collected between 24th of March and 8th of July 2021 using the Lime Survey platform (https://www.limesurvey.org), a secure web-based software designed to securely conduct online surveys. The questionnaire included 58 questions and took around 20 minutes to complete. Although most data collection was done online, data were also collected face-to-face to ensure the participation of vulnerable groups. Face-to-face data collection (*N* = 78) took place at sexual and reproductive health centres, a service for sex workers, and a food bank in the Barcelona area.

### Participants, sampling and recruitment

Participants were women and PWM aged 18–55 who lived in Spain at the time of data collection. Main exclusion criteria were having entered menopause. Participants taking hormonal contraception were excluded from the analyses for this article (*N* = 3465). At least 1535 participants were required, based on sample size calculations. These were performed for the “Equity and Menstrual Health in Spain” project, considering a “menstrual hygiene management” variable. Maximum indetermination of the main variable (proportion of 50%) was assumed. These assumptions were in order to obtain a precision of 2.5% in the confidence intervals. These estimates have been calculated assuming an alfa risk of 5%. PASS software was used for the sample size calculations [PASS 15 Power Analysis and Sample Size Software (2017). NCSS, LLC. Kaysville, Utah, USA]. Sampling was non-probabilistic and purposive. Recruitment strategies included dissemination of the survey in social media, primary healthcare centres, sexual and reproductive healthcare centres, non-governmental and other local organisations. Snowballing techniques were also used.

### Variables

Menstrual characteristics were: age at menarche (≤10; 11–12; 13–15; ≥16), menstrual bleeding abundance (< 25 ml; 25-80 ml; > 80 ml), menstrual blood clots (yes, no), menstrual bleeding duration (< 2 days; 2–7 days; > 7 days), menstrual cycle duration (< 21 days; 21–35 days; > 35 days), menstrual pain (low intensity; moderate intensity; high intensity), menstrual pain management, and premenstrual symptoms (always/many times; some/a few times; never). Reports on premenstrual symptoms were collected through a question on experiences of emotional fluctuations (e.g., sadness or irritability) and physical changes (e.g., tiredness or liquid retention) in the week/2 weeks preceding menstrual bleeding. Gynaecological and systemic health conditions were also collected: anaemia; iron deficiency; uterine myomas; endometrial polyp; endometriosis/adenomyosis; polycystic ovary syndrome; premenstrual syndrome/dysphoric premenstrual disorder; gynaecological cancers (ovary or fallopian tube cancer; uterine cancer; breast cancer); and no diagnoses. Data on menstrual bleeding abundance was collected by asking participants the number of menstrual products used per menstruation [light bleeding, < 25 ml per menstruation (≤6 regular absorbency tampons or pads, or less than 1 full 20 ml menstrual cup); moderate bleeding, 25-80 ml per menstruation (7–15 or 7–19 regular absorbency tampons or pads respectively, or between 1 and 4 full 20 ml menstrual cups); heavy bleeding, > 80 ml per menstruation (≥16 or ≥ 20 regular absorbency tampons or pads respectively, or more than 4 full 20 ml menstrual cups)] [55].

Sociodemographics included: age (18-35, 36-45, 46-55), gender (woman, non-binary/other), trans (yes, don’t know, no), country of birth (Spain; other countries), administrative situation (Spanish nationality; permanent residence; temporal residence; no permit), employment status (working full or part time; studying full or part time; self-employed; unemployed, COVID19 or other benefits; unpaid carer/houseworker), educational attainment (no education, primary education, secondary education, university education), financial constraints < 12 months (always/many times, some/a few times, never), caregiver (yes, no). Self-rated health was categorized using a 5-point Likert scale (excellent, very good, good, fair, poor), based on participants’ responses to the following validated question: *“In general, how would you say your health is?”*

More details on the questionnaire can be found in the Supplementary File 1.

### Data analysis

Descriptive statistics were calculated for each variable to identify asymmetric distributions. Age and age at menarche were analyzed as means (Standard Deviation (SD)) based on the normality of the distribution, and categorical variables were described as percentages. Descriptive statistics were calculated to characterize sociodemographic characteristics, menstrual characteristics, and gynaecological and systemic health conditions. Chi-square tests were used to assess differences between socioeconomic variables, menstrual characteristics and gynaecological and systemic health conditions, according to age. Logistics and multinomial logistic regression models were constructed to compare odds of menstrual characteristics dependent variables (menstrual bleeding abundance, menstrual blood clots, menstrual bleeding duration, menstrual cycle duration, menstrual pain, and premenstrual symptoms) based on independent variables (age, completed education, being a caregiver (yes/no), experiencing financial constraints in the last 12 months (always or many times/some or a few times/never), and self-rated health). Analyses were adjusted by age, educational attainment, financial constraints < 12 months, caregiver and self-rated health. These variables were chosen based on preliminary analyses. Statistical significance was set at 0.05. Analyses were conducted using SPSS 25.0 (SPSS Inc., Armonk, NY: IBM Corp), and Stata/MP 17.0 (StataCorp LLC, TX).

## Results

### Participant characteristics

Data from 19,358 women and PWM were included. Mean age was 33.8 (*SD = 8.7)*. Most identified as women (96.6%) while 3.4% as non-binary/other and 0.8% identified as trans. Also, most participants were born in Spain (93.4%) and held Spanish nationality (95.8%). Over half (65.0%) were working at the time of the research, and 70.7% had completed university studies. 35.3% reported being caregivers of someone else (e.g., children). Almost half reported financial problems in the 12 months prior to the study (42.8%). Most participants indicated their self-rated health to be good (45.9%), very good (38.5%) or excellent (6.5%). See Table 1 for more details.

**Table 1:** Participants’ sociodemographic characteristics, menstrual characteristics, and health conditions (N = 19,358)

Variable	N (%)
**Age**	M (SD) = 33.8 (8.7)
18–25	4155 (21.5%)
26–35	6753 (34.9%)
36–45	6587 (34.0%)
46–55	1863 (9.6%)
**Gender**	
Women	18,706 (96.6%)
Non-binary/other	652 (3.4%)
**Trans**	
Yes	158 (0.8%)
No	19,055 (98.4%)
Don’t know	145 (0.8%)
**Country of birth**	
Spain	17,745 (93.4%)
Other	1264 (6.6%)
**Administrative situation**	
Spanish nationality	18,492 (95.8%)
Permanent residency	619 (3.2%)
Temporal residency	145 (0.8%)
No permit/in process	43 (0.2%)
**Employment situation**	
Working full-time/part-time	12,574 (65.0%)
Self-employed	1834 (9.5%)
Studying full-time/part-time	4600 (23.8%)
Unemployment, COVID-19, retirement and other benefits	1687 (8.7%)
Unpaid carer/houseworker	1020 (5.3%)
**Completed education**	
Primary education	214 (1.1%)
Secondary education	5450 (28.2%)
University education	13,664 (70.7%)
**Caregiver**	
No	12,464 (64.7%)
Yes	6789 (35.3%)
**Financial problems < 12 months**	
Always/Many times	2245 (11.8%)
Some/A few times	5878 (31.0%)
Never	10,847 (57.2%)
**Self-rated health**	
Excellent	1262 (6.5%)
Very good	7430 (38.5%)
Good	8865 (45.9%)
Fair	1603 (8.3%)
Poor	153 (0.8%)
**Age at menarche**	M (SD) = 12.4 (1.5)
≤10	1491 (7.8%)
11–12	9124 (47.8%)
13–15	7956 (41.7%)
≥16	499 (2.6%)
**Menstrual bleeding abundance**	
< 25 ml	3335 (17.2%)
25–80 ml	12,074 (62.4%)
> 80 ml	3923 (20.3%)
**Menstrual blood clots**	
Yes	6957 (35.9%)
No	12,401 (64.1%)
**Menstrual bleeding duration**	
< 2 days	316 (1.6%)
2–7 days	17,765 (92.0%)
> 7 days	1239 (6.4%)
**Menstrual cycle duration**	
< 21 days	1527 (8.0%)
21–35 days	15,829 (83.0%)
> 35 days	1707 (9.0%)
**Menstrual pain**	
Low intensity	5874 (31.0%)
Moderate intensity	8792 (46.3%)
High intensity	4308 (22.7%)
**Menstrual pain management**	
Use of analgesics	12,978 (68.4%)
Use of natural remedies	9886 (52.1%)
Yoga/Meditation or physical activity	160 (0.8%)
Resting, self-care (including through nutrition)	299 (1.6%)
Sex (including masturbation)	59 (0.3%)
Cannabis, cannabidiol (CBD), or alcohol use	44 (0.2%)
Alternative medicine	20 (0.1%)
Accessing the emergency room	14 (0.1%)
Physiotherapy/Osteotherapy	16 (0.1%)
Transcutaneous electric stimulation	7 (0.0%)
Cannot afford menstrual pain management products	40 (0.2%)
I do not do anything	3719 (19.6%)
No menstrual pain	1520 (8.0%)
Do not know what to do	526 (2.8%)
**Premenstrual symptoms**	
Always/Many times	12,009 (68.2%)
Some/A few times	4751 (27.0%)
Never	844 (4.8%)
**Gynaecological and systemic health conditions**	
Anaemia	5958 (30.8%)
Iron deficiency	8601 (44.4%)
Uterine myomas	1454 (7.5%)
Endometrial polyps	989 (5.1%)
Endometriosis/adenomyosis	925 (4.8%)
Polycystic ovary syndrome	3072 (15.9%)
Premenstrual syndrome/dysphoric premenstrual disorder	2629 (13.6%)
Gynaecological cancers (ovary or fallopian tube, uterine and cervical)	107 (0.6%)
Breast cancer	74 (0.4%)
No diagnosis	5704 (29.5%)

The percentages of some variables are above 100% as participants could choose multiple category for some questions

### Menstrual characteristics

**Fig. 1:**
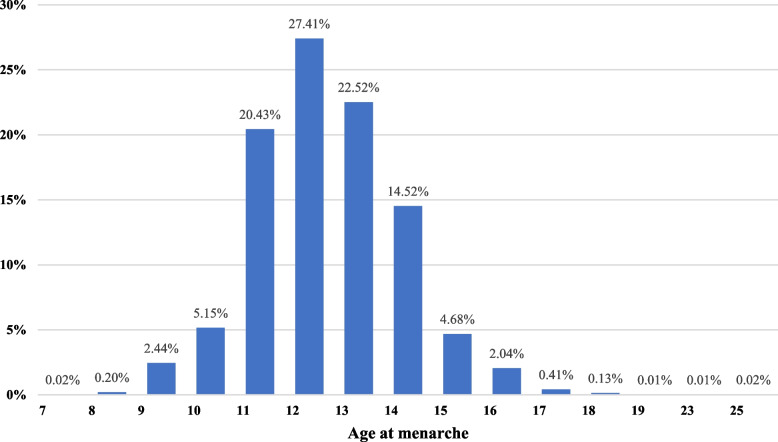
Distribution of age at menarche (*N* = 19,070)

Mean age at menarche was 12.4 (*SD = 1.5*) and was most commonly reported between 11-12 years old (47.8%) and 13–15 years old (41.7%) (see Fig. 1). Menstrual bleeding abundance was between 25 and 80 ml in 62.4% of women and PWM. Over a third indicated having menstrual blood clots (35.9%). The duration of menstrual bleeding was between 2 and 7 days for most (92.0%); 6.4% experienced bleeding for over 7 days. As for menstrual cycle duration, the most common was between 21 and 35 days (83.0%). Moderate intensity menstrual pain was reported by 46.3% of women and PWM, followed by high intensity (22.7%). Menstrual pain was mostly managed by using analgesics (68.4%) and natural remedies (52.1%); 19.6% reported not doing anything to manage menstrual pain. Premenstrual symptoms were experienced always/many times by 68.2% of women and PWM; 27% reported premenstrual symptoms in some or a few menstrual cycles. Self-reports of lifetime gynaecological and systemic health conditions were predominantly of iron deficiency (44.4%), anaemia (30.8%), polycystic ovary syndrome (15.9%), premenstrual syndrome or dysphoric premenstrual disorder (13.6%), uterine myomas (7.5%) and endometriosis or adenomyosis (4.8%). See Table 1 for further information.

When stratifying analyses by age, we observed an age gradient in age at menarche, with early menarche (≤10) and between 11 and 12 years being more common in the 18–25 years old group (9.2 and 51.7%, respectively) than the other age groups, and especially compared to participants aged 46–55 (6.5 and 40.9% respectively). Menstrual bleeding abundance between 25 and 80 ml was most common among younger age groups, while lighter (< 25 ml) and higher (> 80 ml) abundance was more frequently reported as age increased. Longer menstrual bleeding duration (> 7 days) was more commonly indicated by 46–55 (8.8%) and 18–25 (7.1%) aged participants. Shorter bleeding duration (< 2 days) increased with age. Likewise, reports on menstrual cycles shorter than 21 days were higher as age increased, except for the 26–35 age group. Longer menstrual cycles (> 35 days) were most common among participants aged 18–35. Differences in menstrual pain levels were evident by age groups, with high intensity menstrual pain increasing as age decreased (30.2% in 18–25 age group vs 15.0% in 46–55 age group). The use of analgesics and natural remedies for pain management also decreased as age increased. Disparities in premenstrual symptoms reporting by age were unclear and not as pronounced. Moreover, reports of uterine myomas, endometrial polyps and endometriosis significantly increased with age. Self-rated health hardly varied amongst age groups, with poorer health slightly increasing with age. Refer to Table 2 for more details.

**Table 2:** Participants’ sociodemographic characteristics, menstrual characteristics and health conditions, stratified by age (*N* = 19,385)

Variable	Age				*p* value
	18–25	26–35	36–45	46–55	
	N (%)	N (%)	N (%)	N (%)	
**Gender**					
Women	3904 (94.0%)	6537 (96.8%)	6443 (97.8%)	1822 (97.8%)	< 0.001
Non-binary/other	251 (6.0%)	216 (3.2%)	144 (2.2%)	41 (2.2%)	
**Trans**					
Yes	107 (2.6%)	38 (0.6%)	10 (0.2%)	3 (0.2%)	< 0.001
No	3986 (95.9%)	6663 (98.7%)	6553 (99.5%)	1853 (99.5%)	
Don’t know	62 (1.5%)	52 (0.8%)	24 (0.4%)	7 (0.4%)	
**Country of birth**					
Spain	3859 (94.4%)	6232 (93.6%)	5988 (92.8%)	1666 (92.0%)	0.001
Other	231 (5.6%)	423 (6.4%)	466 (7.2%)	144 (8.0%)	
**Administrative situation**					
Spanish nationality	3989 (96.3%)	6428 (95.5%)	6294 (95.8%)	1781 (96.0%)	< 0.001
Permanent residency	102 (2.5%)	209 (3.1%)	241 (3.7%)	67 (3.6%)	
Temporal residency	42 (1.0%)	79 (1.2%)	19 (0.3%)	5 (0.3%)	
No permit/in process	9 (0.2%)	17 (0.3%)	15 (0.2%)	2 (0.1%)	
**Employment situation**					
Working full-time/part-time	1319 (31.7%)	5014 (74.2%)	4811 (73.0%)	1430 (76.8%)	< 0.001
Self-employed	70 (1.7%)	671 (9.9%)	868 (13.2%)	225 (12.1%)	< 0.001
Studying full-time/part-time	3112 (74.9%)	1116 (16.5%)	320 (4.9%)	52 (2.8%)	< 0.001
Unemployment, COVID19, retirement and other benefits	293 (7.1%)	661 (9.8%)	594 (9.0%)	139 (7.5%)	< 0.001
Unpaid carer/houseworker	134 (3.2%)	341 (5.0%)	448 (6.8%)	97 (5.2%)	< 0.001
**Completed education**					
Primary education	40 (1.0%)	29 (0.4%)	93 (1.4%)	52 (2.8%)	< 0.001
Secondary education	2377 (57.3%)	1264 (18.7%)	1333 (20.3%)	476 (25.6%)	
University education	1733 (41.8%)	5450 (80.8%)	5149 (78.3%)	1332 (71.6%)	
**Caregiver**					
No	3987 (96.8%)	5416 (80.7%)	2520 (38.4%)	541 (29.1%)	< 0.001
Yes	130 (3.2%)	1295 (19.3%)	4048 (61.6%)	1316 (70.9%)	
**Financial problems < 12 months**					
Always/Many times	505 (12.8%)	978 (14.7%)	611 (9.4%)	151 (8.2%)	< 0.001
Some/A few times	1399 (35.6%)	2164 (32.4%)	1820 (27.9%)	495 (26.9%)	
Never	2029 (51.6%)	3533 (52.9%)	4090 (62.7%)	1195 (64.9%)	
**Self-rated health**					
Excellent	289 (7.0%)	470 (7.0%)	393 (6.0%)	110 (5.9%)	< 0.001
Very good	1661 (40.4%)	2789 (41.4%)	2387 (36.3%)	593 (31.9%)	
Good	1789 (43.5%)	2948 (43.7%)	3188 (48.5%)	940 (50.6%)	
Fair	374 (9.1%)	492 (7.3%)	550 (8.4%)	187 (10.1%)	
Poor	29 (0.7%)	42 (0.6%)	55 (0.8%)	27 (1.5%)	
**Age at menarche** [M (SD)]	12.2 (1.4)	*12.3 (1.5)*	*12.5 (1.5)*	*12.6 (1.6)*	< 0.001
≤10	372 (9.2%)	540 (8.1%)	460 (7.1%)	119 (6.5%)	< 0.001
11–12	2092 (51.7%)	3299 (49.5%)	2980 (45.7%)	753 (40.9%)	
13–15	1507 (37.2%)	2660 (40.0%)	2894 (44.4%)	895 (48.6%)	
≥16	78 (1.9%)	159 (2.4%)	188 (2.9%)	74 (4.0%)	
**Menstrual bleeding abundance**					
< 25 ml	664 (16.0%)	1198 (17.7%)	1103 (16.7%)	370 (19.9%)	0.001
25–80 ml	2822 (67.9%)	4370 (64.7%)	3894 (59.1%)	988 (53.0%)	< 0.001
> 80 ml	736 (17.7%)	1233 (18.3%)	1513 (23.0%)	441 (23.7%)	< 0.001
**Menstrual blood clots**					
No	2585 (62.2%)	4293 (63.6%)	4284 (65.0%)	1239 (66.5%)	0.002
Yes	1570 (37.8%)	2460 (36.4%)	2303 (35.0%)	624 (33.5%)	
**Menstrual bleeding duration**					
< 2 days	33 (0.8%)	98 (1.5%)	129 (2.0%)	56 (3.0%)	< 0.001
2–7 days	3820 (92.1%)	6252 (92.7%)	6061 (92.1%)	1632 (88.2%)	
> 7 days	294 (7.1%)	395 (5.9%)	388 (5.9%)	162 (8.8%)	
**Menstrual cycle duration**					
< 21 days	311 (7.7%)	437 (6.6%)	588 (9.0%)	191 (10.6%)	< 0.001
21–35 days	3158 (78.1%)	5534 (83.1%)	5686 (86.8%)	1451 (80.2%)	
> 35 days	574 (14.2%)	690 (10.4%)	275 (4.2%)	168 (9.3%)	
**Menstrual pain**					
Low intensity (1–3)	733 (18.1%)	1677 (25.3%)	2574 (39.8%)	890 (48.8%)	< 0.001
Moderate intensity (4–7)	2102 (51.8%)	3245 (49.0%)	2784 (43.0%)	661 (36.2%)	
High intensity (8–10)	1224 (30.2%)	1700 (25.7%)	1111 (17.2%)	273 (15.0%)	
**Menstrual pain management**					
Use of analgesics	3079 (75.9%)	4607 (69.6%)	4167 (64.4%)	1125 (61.6%)	< 0.001
Use of natural remedies	2458 (60.6%)	3999 (60.4%)	2861 (44.2%)	568 (31.1%)	< 0.001
Yoga/Meditation or physical activity	24 (0.6%)	71 (1.1%)	55 (0.8%)	10 (0.5%)	0.027
Resting, self-care (including through nutrition)	34 (0.8%)	97 (1.5%)	141 (2.2%)	24 (1.5%)	< 0.001
Sex (including masturbation)	8 (0.2%)	30 (0.5%)	19 (0.3%)	2 (0.1%)	0.037
Cannabis, cannabidiol (CBD), or alcohol use	6 (0.1%)	24 (0.4%)	13 (0.2%)	1 (0.1%)	0.031
Alternative medicine	2 (0.0%)	19 (0.3%)	20 (0.3%)	3 (0.2%)	0.033
Accessing the emergency room	6 (0.1%)	4 (0.1%)	2 (0.0%)	2 (0.1%)	0.164
Physiotherapy/Osteotherapy	1 (0.0%)	5 (0.1%)	9 (0.1%)	1 (0.1%)	0.234
Transcutaneous electric stimulation	1 (0.0%)	4 (0.1%)	2 (0.0%)	0 (0.0%)	0.595
Cannot afford menstrual pain management products	17 (0.4%)	13 (0.2%)	7 (0.1%)	3 (0.2%)	0.008
I do not do anything	823 (20.3%)	1176 (17.8%)	1342 (20.7%)	378 (20.7%)	< 0.001
I experience no menstrual pain	218 (5.4%)	422 (6.4%)	642 (9.9%)	238 (13.0%)	< 0.001
I do not know what to do	200 (4.9%)	227 (3.4%)	88 (1.4%)	11 (0.6%)	< 0.001
**Premenstrual symptoms**					
Always/Many times	2552 (68.2%)	4331 (70.5%)	4068 (67.7%)	1058 (61.9%)	< 0.001
Some/A few times	1011 (27.0%)	1562 (25.4%)	1641 (27.3%)	537 (31.4%)	
Never	179 (4.8%)	250 (4.1%)	302 (5.0%)	113 (6.6%)	
**Gynecological and systemic health conditions**					
Anemia	987 (23.8%)	2047 (30.3%)	2287 (34.7%)	637 (34.2%)	< 0.001
Iron deficiency	1413 (34.0%)	2860 (42.4%)	3355 (50.9%)	973 (52.2%)	< 0.001
Uterine myomas	17 (0.4%)	234 (3.5%)	786 (11.9%)	417 (22.4%)	< 0.001
Endometrial polyps	43 (1.0%)	222 (3.3%)	502 (7.6%)	222 (11.9%)	< 0.001
Endometriosis/adenomyosis	60 (1.4%)	307 (4.5%)	421 (6.4%)	137 (7.4%)	< 0.001
Polycystic ovary syndrome	464 (11.2%)	1312 (19.4%)	1089 (16.5%)	207 (11.1%)	< 0.001
Premenstrual syndrome/dysphoric premenstrual disorder	410 (9.9%)	952 (14.1%)	971 (14.7%)	296 (15.9%)	< 0.001
Gynaecological cancers (ovary or fallopian tube, urine and cervical)	2 (0.0%)	37 (0.5%)	54 (0.8%)	14 (0.8%)	< 0.001
Breast cancer	1 (0.0%)	6 (0.1%)	36 (0.5%)	31 (1.7%)	< 0.001
No diagnosis	1846 (44.4%)	1983 (29.4%)	1463 (22.2%)	412 (22.1%)	< 0.001

### Associations between menstrual characteristics, sociodemographics and self-rated health

An age gradient was identified for menstrual abundance, with the odds of light menstrual flow (< 25 ml) being significantly higher among participants aged 46–55 (aOR: 1.56, 95% CI, 1.33–1.83). Similarly, light menstruations were more common as educational attainment decreased (eg., aOR_primary education_: 1.86, 95% CI, 1.35–2.57). The odds for menstrual abundance over 80 ml also decreased with education (eg., aOR_primary education_: 0.68, 95% CI, 0.47–0.98). The odds for abundant menstrual flow (> 80 ml) were significantly higher among caregivers (aOR: 1.38, 95% CI, 1.27–1.51). However, caregivers were less likely to report menstrual blood clots (aOR: 0.86, 95% CI, 0.80–0.93). A gradient was also identified regarding financial problems in the 12 months preceding data collection, as more severe financial difficulties were significantly associated with higher odds for abundant menstrual flow (> 80 ml) (aOR: 1.19, 95% CI, 1.06–1.34) and menstrual blood clots (aOR: 1.21, 95% CI, 1.10–1.33). The odds for a light menstrual flow (< 25 ml) decreased as self-rated health worsened (aOR_fair self-rated health_: 0.64, 95% CI, 0.53–0.79), except for poor self-rated health. In turn, the odds for abundant menstrual flow (> 80 ml) (aOR_poor self-rated health_: 2.08, 95% CI, 1.43–3.01), and menstrual blood clots (aOR_poor self-rated health_: 2.90, 95% CI, 2.05–4.10) were higher as self-rated health worsened. See Table 3 for more details.

**Table 3:** Associations between menstrual bleeding abundance, menstrual blood clots, sociodemographic characteristics and self-rated health (*N* = 18,839)

	Menstrual bleeding abundance*						Menstrual blood clots*	
	< 25 ml		25–80 ml		> 80 ml			
	aOR (95%CI)	*p* value	aOR (95%CI)	*p* value	aOR (95%CI)	*p* value	aOR (95%CI)	*p* value
**Age**								
18–25	1.00		1.00		1.00		1.00	
26–35	1.29 (1.15–1.45)	< 0.001	0.84 (0.77–0.92)	< 0.001	0.96 (0.86–1.07)	0.418	0.94 (0.87–1.03)	0.205
36–45	1.28 (1.13–1.45)	< 0.001	0.72 (0.65–0.79)	< 0.001	1.12 (0.99–1.26)	0.067	0.94 (0.86–1.04)	0.242
46–55	1.56 (1.33–1.83)	< 0.001	0.58 (0.51–0.66)	< 0.001	1.13 (0.97–1.31)	0.125	0.89 (0.78–1.02)	0.084
**Completed education**								
University education	1.00		1.00		1.00		1.00	
Secondary education	1.32 (1.21–1.45)	< 0.001	0.87 (0.81–0.93)	< 0.001	0.96 (0.88–1.05)	0.365	0.97 (0.90–1.04)	0.416
Primary education	1.86 (1.35–2.57)	< 0.001	0.64 (0.49–0.85)	0.002	0.68 (0.47–0.98)	0.038	0.82 (0.61–1.11)	0.200
**Caregiver**								
No	1.00		1.00		1.00		1.00	
Yes	0.88 (0.80–0.97)	0.008	0.84 (0.78–0.90)	< 0.001	1.38 (1.27–1.51)	< 0.001	0.86 (0.80–0.93)	< 0.001
**Financial problems < 12 months**								
Never	1.00		1.00		1.00		1.00	
Some/A few times	1.03 (0.94–1.12)	0.528	0.97 (0.91–1.04)	0.464	1.04 (0.95–1.12)	0.398	1.08 (1.01–1.15)	0.035
Always/Many times	1.08 (0.96–1.22)	0.219	0.84 (0.76–0.93)	0.001	1.19 (1.06–1.34)	0.003	1.21 (1.10–1.33)	< 0.001
**Self-rated health**								
Excellent	1.00		1.00		1.00		1.00	
Very good	0.83 (0.72–0.97)	0.019	1.13 (0.99–1.27)	0.064	0.98 (0.84–1.15)	0.839	1.24 (1.08–1.41)	0.002
Good	0.77 (0.66–0.89)	0.001	1.03 (0.91–1.16)	0.649	1.15 (0.99–1.34)	0.075	1.51 (1.33–1.73)	< 0.001
Fair	0.64 (0.53–0.79)	< 0.001	0.94 (0.81–1.10)	0.450	1.51 (1.25–1.81)	< 0.001	2.05 (1.74–2.40)	< 0.001
Poor	0.73 (0.47–1.15)	0.172	0.49 (0.35–0.70)	< 0.001	2.08 (1.43–3.01)	< 0.001	2.90 (2.05–4.10)	< 0.001

* < 25 ml, 25-80 ml, and > 80 ml are three separate variables with yes/no values, as a participant may have a ‘yes’ value in one or more of these variables. All the models presented in this table are logistic regression models

As reported in Table 4, the odds for short menstruation duration (< 2 days) (aOR_age 46–55_: 4.80, 95% CI, 2.91–7.93) and menstrual cycles (< 21 days) (aOR_age 46–55_: 1.47, 95% CI, 1.18–1.83) were higher as age increased. A similar gradient was found by completed education; menstrual duration of 2 days or less (aOR_primary education_: 2.87, 95% CI, 1.42–5.79) and menstrual cycles that were 21 days or shorter (aOR_primary education_: 2.97, 95% CI, 2.06–4.28) were more likely among participants with completed primary education. The odds for long menstruations (> 7 days) were also higher as educational attainment decreased (aOR_primary education_: 2.05, 95% CI, 1.35–3.11). Caregivers had higher odds for reporting menstrual duration of 7 days or over (aOR: 1.24, 95% CI, 1.07–1.43). The odds for long menstruations (aOR_always/many times_: 1.45, 95% CI, 1.22–1.74) and short menstrual cycles (aOR_always/many times_: 1.43, 95% CI, 1.21–1.68) increased as women and PWM reported more financial difficulties. Poor self-rated health was also associated with shorter (aOR: 2.89, 95% CI, 1.30–6.39) and longer (aOR: 6.23, 95% CI, 3.70–10.47) menstruations, and shorter (aOR: 2.15, 95% CI, 1.30–3.55) and longer (aOR: 1.89, 95% CI, 1.10–3.23) menstrual cycles.

**Table 4:** Associations between menstrual bleeding duration, menstrual cycle duration, sociodemographic characteristics, and self-rated health

	Menstrual bleeding duration* N = 18,818					Menstrual cycle duration* N = 18,581				
	< 2 days		2–7 days	> 7 days		< 21 days		21–35 days	> 35 days	
	aOR (95%CI)	*p* value	Ref	aOR (95%CI)	*p* value	aOR (95%CI)	*p* value	Ref	aOR (95%CI)	*p* value
**Age**										
18–25	1.00			1.00		1.00			1.00	
26–35	2.25 (1.45–3.51)	< 0.001		0.82 (0.69–0.98)	0.026	0.98 (0.83–1.16)	0.801		0.69 (0.60–0.79)	< 0.001
36–45	3.12 (1.99–4.91)	< 0.001		0.77 (0.64–0.94)	0.009	1.23 (1.03–1.46)	0.022		0.27 (0.23–0.32)	< 0.001
46–55	4.80 (2.91–7.93)	< 0.001		1.15 (0.91–1.46)	0.231	1.47 (1.18–1.83)	0.001		0.64 (0.52–0.79)	< 0.001
**Completed education**										
University education	1.00			1.00		1.00			1.00	
Secondary education	1.49 (1.14–1.95)	0.003		1.15 (1.00–1.32)	0.052	1.70 (1.51–1.93)	< 0.001		0.98 (0.86–1.10)	0.714
Primary education	2.87 (1.42–5.79)	0.003		2.05 (1.35–3.11)	0.001	2.97 (2.06–4.28)	< 0.001		1.00 (0.57–1.75)	0.993
**Caregiver**										
No	1.00			1.00		1.00			1.00	
Yes	1.02 (0.78–1.33)	0.893		1.24 (1.07–1.43)	0.004	1.09 (0.96–1.25)	0.179		0.92 (0.80–1.05)	0.221
**Financial problems < 12 months**										
Never	1.00			1.00		1.00			1.00	
Some/A few times	1.06 (0.81–1.38)	0.681		1.25 (1.09–1.43)	0.001	1.31 (1.16–1.48)	< 0.001		1.01 (0.90–1.14)	0.821
Always/Many times	1.19 (0.83–1.70)	0.352		1.45 (1.22–1.74)	< 0.001	1.43 (1.21–1.68)	< 0.001		1.07 (0.91–1.26)	0.428
**Self-rated health**										
Excellent	1.00			1.00		1.00			1.00	
Very good	0.70 (0.45–1.08)	0.103		1.56 (1.13–2.17)	0.008	0.91 (0.72–1.15)	0.445		0.85 (0.68–1.05)	0.128
Good	0.67 (0.44–1.03)	0.067		1.97 (1.42–2.72)	< 0.001	1.02 (0.81–1.28)	0.876		1.08 (0.88–1.34)	0.469
Fair	0.89 (0.52–1.52)	0.675		3.08 (2.16–4.39)	< 0.001	1.29 (0.98–1.70)	0.069		1.59 (1.24–2.05)	< 0.001
Poor	2.89 (1.30–6.39)	0.009		6.23 (3.70–10.47)	< 0.001	2.15 (1.30–3.55)	0.003		1.89 (1.10–3.23)	0.021

*Multinomial logistic regression models

Odds for moderate (aOR_aged 46–55_: 0.36, 95% CI, 0.31–0.42) and high (aOR_aged46–55_: 0.33, 95% CI, 0.27–0.40) intensity menstrual pain decreased as age increased. They were also lower among caregivers (aOR_high intensity pain_: 0.39, 95% CI, 0.35–0.44). Instead, the odds for both moderate (aOR_always/many times_: 1.21, 95% CI, 1.07–1.37) and high (aOR_always/many times_: 1.87, 95% CI, 1.63–2.14) intensity menstrual pain were higher as participants reported more financial problems in the 12 months preceding the study. A gradient was also found regarding self-rated health. The worse health was perceived, the higher the odds for moderate (aOR_poor self-rated health_: 2.70, 95% CI, 1.61–4.52) and high (aOR_poor self-rated health_: 8.33, 95% CI, 4.97–13.94) intensity menstrual pain. See Table 5 for further details.

**Table 5:** Associations between menstrual pain, sociodemographic characteristics, and self-rated health (N = 18,467)

	Menstrual pain*				
	Low intensity	Moderate intensity		High intensity	
	Ref	aOR (95%CI)	*p* value	aOR (95%CI)	*p* value
**Age**					
18–25		1.00		1.00	
26–35		0.75 (0.67–0.84)	< 0.001	0.74 (0.65–0.84)	< 0.001
36–45		0.50 (0.45–0.57)	< 0.001	0.44 (0.39–0.51)	< 0.001
46–55		0.36 (0.31–0.42)	< 0.001	0.33 (0.27–0.40)	< 0.001
**Completed education**					
University education		1.00		1.00	
Secondary education		1.06 (0.97–1.15)	0.200	1.19 (1.07–1.31)	<0.001
Primary education		1.04 (0.75–1.45)	0.815	1.10 (0.73–1.65)	<0.645
**Caregiver**					
No		1.00		1.00	
Yes		0.61 (0.56–0.66)	< 0.001	0.39 (0.35–0.44)	< 0.001
**Financial problems < 12 months**					
Never		1.00		1.00	
Some/A few times		1.21 (1.12–1.31)	< 0.001	1.46 (1.33–1.61)	< 0.001
Always/Many times		1.21 (1.07–1.37)	0.002	1.87 (1.63–2.14)	< 0.001
**Self-rated health**					
Excellent		1.00		1.00	
Very good		1.39 (1.21–1.60)	< 0.001	1.41 (1.18–1.68)	< 0.001
Good		1.88 (1.64–2.16)	< 0.001	2.00 (1.67–2.38)	< 0.001
Fair		2.63 (2.17–3.18)	< 0.001	4.36 (3.49–5.45)	< 0.001
Poor		2.70 (1.61–4.52)	< 0.001	8.33 (4.97–13.94)	< 0.001

*Multinomial logistic regression models

Lastly, being a caregiver appeared to be a protective factor for experiencing premenstrual symptoms always/many times (aOR: 0.60, 95% CI, 0.50–0.71) and sometimes (aOR: 0.70, 95% CI, 0.58–0.84). On the other hand, risk factors were reporting financial difficulties (< 12 months) (aOR_always/many times_: 2.80, 95% CI, 2.06–3.79) and worsened self-rated health (aOR_poor self-rated health_: 3.34, 95% CI, 1.20–9.31). Refer to Table 6 for more information.

**Table 6:** Associations between premenstrual symptoms, sociodemographic characteristics, and self-rated health (*N* = 17,158)

	Premenstrual symptoms*				
	Always/many times		Some/a few times		Never
	aOR (95%CI)	*p* value	aOR (95%CI)	*p* value	Ref
**Age**					
18–25	1.00		1.00		
26–35	1.21 (0.97–1.51)	0.085	1.13 (0.90–1.43)	0.290	
36–45	1.23 (0.97–1.57)	0.093	1.17 (0.91–1.51)	0.211	
46–55	0.89 (0.66–1.19)	0.415	1.04 (0.77–1.41)	0.802	
**Completed education**					
University education	1.00		1.00		
Secondary education	0.82 (0.69–0.98)	0.030	0.89 (0.74–1.07)	0.228	
Primary education	0.90 (0.41–1.96)	0.789	1.20 (0.54–2.67)	0.656	
**Caregiver**					
No	1.00		1.00		
Yes	0.60 (0.50–0.71)	< 0.001	0.70 (0.58–0.84)	< 0.001	
**Financial problems < 12 months**					
Never	1.00		1.00		
Some/A few times	2.06 (1.72–2.47)	< 0.001	1.63 (1.35–1.96)	< 0.001	
Always/Many times	2.80 (2.06–3.79)	< 0.001	1.48 (1.08–2.04)	0.015	
**Self-rated health**					
Excellent	1.00		1.00		
Very good	1.50 (1.18–1.91)	0.001	1.38 (1.08–1.78)	0.011	
Good	2.40 (1.88–3.06)	< 0.001	1.81 (1.40–2.34)	< 0.001	
Fair	4.66 (3.02–7.20)	< 0.001	2.53 (1.61–3.98)	< 0.001	
Poor	3.34 (1.20–9.31)	0.021	1.22 (0.40–3.69)	0.722	

*Multinomial logistic regression models

## Discussion

This study aimed to investigate the associations between menstrual characteristics, sociodemographic factors and self-rated health among adult women and PWM in Spain. In our study, age at menarche was 12.4 (SD = 1.5) and most commonly reported between 11 and 12 (47.8%) and 13–15 (41.7%) years old. Menarche was reported before the age of 10 in 7.8% of our participants. These results are consistent with previous evidence, both in Spain [20, 56] and other countries [57–59]. As participants’ age decreased menarche was reported to be at an earlier age, supporting the already evidenced decline in the onset of menstruation since the second half of the twentieth century [31, 32, 60–62]. The reason why age at menarche and its well-established onset decline matters, lays on the implications of early puberty. These include a higher risk of cardiovascular disease, mediated by increased body fat and other mechanisms [63], breast cancer [64, 65], and the emotional and social impact of early menarche [66, 67]. Moreover, it is imperative that the latter implications are further considered, especially as menarche can be understood as a rite of passage to adulthood, which often leads to the sexualization of girls and young menstruators and the constriction of the social and physical spaces they occupy in our and other sociocultural contexts [66].

On the other hand, 37.6% of women and PWM in our study reported menstrual abundance that could not be considered within healthy parameters; 17.2% bled less than 25 ml and 20.3% indicated bleeding more than 80 ml per menstruation. There were also common reports in our study regarding the presence of menstrual blood clots (35.9%) and, although not as frequent, menstrual durations longer than 7 days (6.4%). Heavy menstrual bleeding can be caused by processes interfering endocrine, paracrine and hemostatic functions of the endometrium and the myometrial contractility (e.g., endometrial polyps, adenomyosis, leiomyomas, coagulopathy, hyperplasia, polycystic ovarian syndrome) [68]. However, light menstrual bleeding sometimes is not clearly attributed to a specific cause [69]. Reports of heavy bleeding and menstrual blood clots appear to be lower than those identified in previous research [18, 70, 71]. This may be explained as the lack of access and adequacy of menstrual education considerably limit the resources of women and PWM to identify menstrual health factors (e.g., their bleeding patterns) [1, 2]. Although parameters to calculate menstrual bleeding through calculating the number of menstrual products used were provided to participants in our study, future research should consider alternative ways of measuring bleeding patterns [55]. Heavy bleeding patterns may not only have important health implications, such as in the development of iron deficiency and anemia [72–74], but can have an impact on quality of life [75] and menstrual management. While managing menstruation can be generally challenging, mainly due to structural factors rooted in sociocultural androcentric perspectives and practices, heavy bleeders may encounter increased difficulties. For instance, these challenges may encompass needing adequate facilities in public spaces and changing menstrual products more often than other women and PWM. Considering that the lack of access to menstrual management spaces is a reality for most women and PWM in our study [2] and other contexts [76, 77], it is imperative to explore the health and social implications of heavy bleeding (especially in public spaces) [76], and respond to the menstrual needs of women and PWM through research, advocacy, and policymaking [78].

A recent systematic literature review and meta-analysis including 38 studies conducted in a variety of countries from the Global South and North has identified the prevalence of dysmenorrhea to be 71.1% [79]. In our research, moderate and high intensity menstrual pain reports were also significantly high (46.3 and 22.7%, respectively). Menstrual pain, often caused by hyper-production of uterine prostaglandins, leads to elevated uterine tone and high uterine contractions. Although endocrine factors contribute to menstrual pain, other factors (e.g., age, childbearing, family history of dysmenorrhea, and mental health) play a role in the perception and the severity of pain [80, 81]. For example, childbearing is associated with reduced menstrual pain. Uterine neurotransmitters dynamics change during pregnancy, and after that process, there is a partial regeneration of uterine nerve terminals that may explain the disappearance or reduction of menstrual pain after childbirth [82, 83]. On the other hand, menstrual pain is still systematically normalized and often dismissed, even in healthcare settings [2, 16, 17, 83, 84], which may lead to delays on diagnosis of health conditions (e.g. endometriosis) and a poor quality of life [2, 17], added to the emotional and social implications of experiencing pain [85]. The lack of a structural and social awareness of what a healthy menstrual cycle and menstruation may be has an impact on the few resources that many women and PWM have when it comes to dealing with pain management. Based on our results, menstrual pain was mostly managed by using analgesics (68.4%) and natural remedies (52.1%), while 19.6% reported not doing anything to manage it. Despite the wide variety of methods that can be used and be effective (e.g., physical activity) [86], medicalization is usually the most common strategy within healthcare services [87], especially via hormonal contraceptives or painkillers [2, 88]. Narrowing down the options for menstrual pain management can greatly contribute to pathologizing the menstrual cycle and menstruation [89], rather than considering menstruation and the menstrual cycle as indicators of health [90, 91].

Consistent with another study in the Spanish context [92], premenstrual symptoms reports were also high, since these were experienced by most women and PWM (68.2%) in most menstrual cycles. However, only 13.6% indicated a diagnosis of premenstrual syndrome or dysphoric premenstrual disorder. This may potentially be due to the normalization of premenstrual symptomatology among women, PWM and healthcare professionals. It also points towards the need to attend to premenstrual symptoms regardless of whether they fulfil a diagnostic criterion or not. Although these results do not provide enough information to assess to what extent these symptoms affect participants day-to-day, it is relevant to point towards the potential impact of premenstrual experiences on emotional and social health experienced by women and PWM. As for menstrual pain and other menstrual experiences, healthcare systems and professionals have often not paid enough consideration to premenstrual symptoms. One of the reasons for this is the ingrained stigmatization of menstruation [93] and “the menstruating woman”, portraited as irrational and monstruous [94–96]. The assumption that the bodies of women and PWM are pathological and tend to irrational emotions may have led to underestimation, minimization, and invalidation of (pre)menstrual experiences, maintaining the normalization of pain or fatigue, among other symptoms [95].

The estimated prevalence of self-reported gynaecological and systemic health conditions differs from previous evidence, since women and PWM taking hormonal contraception at the data collection were excluded from the analyses. This links with the abovementioned medicalization of menstrual related health issues [2, 87], frequently treated by default with hormonal contraception [87, 88]. Alternative approaches (e.g., natural remedies, nutrition, or physical activity) are rarely offered, partially as their adequacy and efficacy is often unknown by healthcare professionals, perpetuating a medication-based model to address menstrual issues [2, 89, 97]. Another explanation could be due to the lack of time health professionals often have to approach menstrual health in a more holistic way and to focus on menstrual education. This may however have important implications, as the neglect of menstrual-related symptomatology and its medicalisation are associated with late diagnosis and treatment of health conditions such as endometriosis [98] or ovarian cancer [99].

While the evidence on menstrual health and equity is growing, it is imperative to incorporate a critical perspective on how gender and other social inequities mediate and impact menstrual experiences, health, and equity [2]. Hence, beyond describing menstrual characteristics, this article aimed at identifying the associations between self-reported menstrual patterns and sociodemographic factors that represent axes of social inequities (i.e., age, educational attainment, caregiving, and financial situation). Other axes of inequity (i.e., gender identity, identification as trans, employment status, administrative status, and country of birth) were considered in primary analyses. However, preliminary findings were unsupportive of including these variables in further analyses. A potential reason could be the limited sample size available for certain participant groups (e.g., trans menstruators or those with no permit to reside in Spain). Despite these variables could not be included in our analyses, further research should actively investigate the associations of these axes of inequity with menstrual patterns. Intersectionality approaches could be particularly helpful to highlight social inequities of menstrual health [100].

An age gradient was observed for several menstrual experiences related to pain, bleeding abundance and menstrual cycle’s duration. As expected, the odds for lighter menstrual flow, shorter bleeding days and menstrual cycles, and moderate/high intensity pain were higher as participants were younger. This may be explained by ovarian maturation and low progesterone levels at a younger age. Elevated prostaglandin and diminished progesterone levels contribute to the inflammatory responses, triggering pain in the endometrium [101]. The shortening of menstrual cycles and bleeding duration among those over 40 years old can be explained by the decrease in oestrogen levels and diminished ovarian reserve [102].

Together with other axes of inequity, socioeconomic status is a well-known determinant of health [4, 5], including of menstrual health [103, 104]. In our research and previous literature, reporting financial hardship and lower educational attainment were risk factors for potentially unhealthy menstrual patterns [103]. Reporting financial constraints was associated with abundant flow (> 80 ml), blood clots, long menstruations (> 7 bleeding days), short menstrual cycles (< 21 days), moderate and high intensity pain, and premenstrual symptoms. As in our study, evidence has reported that heavy menstrual bleeding and menstrual pain can be associated with low socioeconomic status. Financial constraints can influence inadequate nutritional status, ultimately affecting menstrual cycle patterns [103]. Risk for light menstruations (< 25 ml), less bleeding days, short menstrual cycles (< 21 days) and menstruations that last over 7 days was higher among participants with lower educational attainment. Lower educational attainment tends to correlate with more precarious employment situations and elevated stress levels. Disruptions in hormonal equilibrium triggered by stress may result in changes in menstrual patterns [105]. These findings suggest the inherent relationship between social inequities and menstrual health and reinforce the need to deeply explore how socioeconomic contexts and stressors may have an impact on menstrual patterns and health (2,9106).

An interesting finding was the role of identifying as a caregiver. Caregivers presented higher odds in abundant menstrual flow and longer menstruation days, which is consistent with previously evidence on caregiving as a factor of impaired health (e.g., mental health, chronic pain) [106, 107]. However, caregiving was also found to be a protective factor for menstrual clots, reporting moderate and high intensity menstrual pain, and experiencing premenstrual symptoms. Caregivers’ age could explain these findings, as most informal caregivers in our study were between 26 and 55 years old. A complementary explanation could be related to a lack of awareness among participants on how to identify menstrual clots, although this could certainly apply to all participants in our study. In addition, the burden of care may limit embodied spaces and awareness to identify and validate experiences of pain and premenstrual symptoms (e.g., tiredness). Future gender-based research could investigate the complex intersections of care work and menstrual health.

On the other hand, and as already stated, self-rated health is a widely used proxy for general health status and health inequities [47–50]. Our data highlight a gradient between poorer self-rated health and higher odds for reporting abundant bleeding (> 80 ml), menstrual blood clots, short (< 21 days) and long (> 35 days) menstrual cycles, moderate and high intensity pain, and premenstrual symptoms. Therefore, these findings are suggestive of a link between general health status and poorer menstrual health patterns. In addition, they strengthen the inherent interconnection between social inequities and menstrual health.

### Strengths and limitations

Main strengths of this research include its social relevance and innovation, as it pioneers in providing evidence on menstrual health and equity in Spain. Another strength is the large sample size included that despite not being representative to the population living in Spain, it includes women and PWM across the whole Spanish territory. Main limitations encompass the study not being representative of the menstruating populations living in Spain and the impact of the digital divide amongst vulnerable and hard-to-reach populations. Recall biases may be present in self-reported variables owing to the retrospective design of the study. Furthermore, using the amount of menstrual products used to determine the abundance of menstrual bleeding may be a limitation, as the frequency of menstrual product change can be influenced by other variables.

## Conclusions

This study presents a detailed overview of menstrual characteristics among adult women and PWM in Spain. The odds for heavy menstrual bleeding, moderate/high intensity menstrual pain and experiencing premenstrual symptoms, among other menstrual characteristics, were higher in participants with less educational attainment, more financial hardship, and poorer self-rated health. In turn, increased age and identifying as a caregiver may be protective factors. This suggests the need to consider how social inequities may impact menstrual health, and the implications for menstrual management. Research highlighting the needs of vulnerable populations is imperative, alongside community-based actions and evidence-based policymaking. Menstrual inequities should be considered and addressed within interventions and public policies, considering menstruation as a vital sign of health and menstrual health as a public health issue. Adequate training to healthcare professionals is essential so that they have enough support to address menstrual health, attending to social inequities of health.

### Supplementary Information

**Additional file 1.**

## Data Availability

The datasets generated and analysed during the current study are not publicly available for confidentiality and anonymity reasons but are available from the corresponding author on reasonable request.

## Abbreviations

PWM: People who menstruate

## References

[1] Hennegan J, Winkler IT, Bobel C, Keiser D, Hampton J, Larsson G (2021). Menstrual health: a definition for policy, practice, and research. Sex Reprod Health Matters..

[2] Holst AS, Jacques-Aviñó C, Berenguera A, Pinzón-Sanabria D, Valls-Llobet C, Munrós-Feliu J (2022). Experiences of menstrual inequity and menstrual health among women and people who menstruate in the Barcelona area (Spain): a qualitative study. Reprod Health..

[3] Weiss-Wolf J (2017). Periods Gone Public. Taking a stand for menstrual equity.

[4] Pickett KE, Wilkinson RG (2015). Income inequality and health: A causal review. Soc Sci Med..

[5] Pascual-Sáez M, Cantarero-Prieto D, Lanza-León P (2019). The dynamics of health poverty in Spain during the economic crisis (2008–2016). Health Policy (New York)..

[6] Bastos A, Casaca SF, Nunes F, Pereirinha J (2009). Women and poverty: A gender-sensitive approach. J Socio Econ..

[7] Sommer M, Phillips-Howard PA, Gruer C, Schmitt ML, Nguyen AM, Berry A (2022). Menstrual product insecurity resulting from COVID-19–related income loss, United States, 2020. Am J Public Health..

[8] Boyers M, Garikipati S, Biggane A, Douglas E, Hawkes N, Kiely C (2022). Period poverty: the perceptions and experiences of impoverished women living in an inner-city area of Northwest England. PLoS One..

[9] Sommer M, Gruer C, Smith RC, Maroko A, Hopper K (2020). Menstruation and homelessness: challenges faced living in shelters and on the street in new York City. Health Place..

[10] Schmitt ML, Clatworthy D, Ratnayake R, Klaesener-Metzner N, Roesch E, Wheeler E (2017). Understanding the menstrual hygiene management challenges facing displaced girls and women: findings from qualitative assessments in Myanmar and Lebanon. Confl Health..

[11] Soeiro RE, Rocha L, Surita FG, Bahamondes L, Costa ML (2021). Period poverty: menstrual health hygiene issues among adolescent and young Venezuelan migrant women at the northwestern border of Brazil. Reprod Health..

[12] Gómez-Acebo I, Dierssen-Sotos T, Palazuelos C, Castanõ-Vinyals G, Pérez-Gómez B, Amiano P (2020). Changes in individual and contextual socio-economic level influence on reproductive behavior in Spanish women in the MCC-Spain study. BMC Womens Health..

[13] Cardoso LF, Scolese AM, Hamidaddin A, Gupta J (2021). Period poverty and mental health implications among college-aged women in the United States. BMC Womens Health..

[14] Gouvernet B, Sebbe F, Chapillon P, Rezrazi A, Brisson J (2022). Period poverty and mental health in times of covid-19 in France. Health Care Women Int..

[15] Rossouw L, Ross H (2021). Understanding Period Poverty: Socio-Economic Inequalities in Menstrual Hygiene Management in Eight Low- and Middle-Income Countries. Int J Environ Res Public Health..

[16] Abreu-Sánchez A, Parra-Fernández ML, Onieva-Zafra MD, Ramos-Pichardo JD, Fernández-Martínez E (2020). Type of Dysmenorrhea, Menstrual Characteristics and Symptoms in Nursing Students in Southern Spain. Healthcare..

[17] Fernández-Martínez E, Fernández-Villa T, Amezcua-Prieto C, Suárez-Varela MM, Mateos-Campos R, Ayán-Pérez C (2020). Menstrual Problems and Lifestyle among Spanish University Women. Int J Environ Res Public Health.

[18] Ansong E, Arhin SK, Cai Y, Xu X, Wu X (2019). Menstrual characteristics, disorders and associated risk factors among female international students in Zhejiang Province, China: a cross-sectional survey. BMC Womens Health..

[19] Santos IS, Minten GC, Valle NCJ, Tuerlinckx GC, Silva AB, Pereira GAR (2011). Menstrual bleeding patterns: A community-based cross-sectional study among women aged 18-45 years in southern Brazil. BMC Womens Health..

[20] Salces I, Rebato EM, Susanne C, San Martin L, Rosique J. Familial resemblance for the age at menarche in Basque population. Ann Hum Biol. 2001;28(2):143–56. 10.1080/03014460151056338 11293723

[21] Karapanou O, Papadimitriou A (2010). Determinants of menarche. Reprod Biol Endocrinol..

[22] Freedman DS, Khan LK, Serdula MK, Dietz WH, Srinivasan SR, Berenson GS (2002). Relation of age at menarche to race, time period, and anthropometric dimensions: the Bogalusa heart study. Pediatrics..

[23] Valls-Llobet C (2018). Medio ambiente y salud. Mujeres y hombres en un mundo de nuevos riesgos.

[24] Lee JE, Jung HW, Lee YJ, Lee YA (2019). Early-life exposure to endocrine-disrupting chemicals and pubertal development in girls. Ann Pediatr Endocrinol Metab..

[25] Özen S, Darcan Ş (2011). Effects of environmental endocrine disruptors on pubertal development. J Clin Res Pediatr Endocrinol..

[26] Canelón SP, Boland MR (2020). A systematic literature review of factors affecting the timing of menarche: the potential for climate change to impact Women’s health. Int J Environ Res Public Health..

[27] Ellis BJ, Garber J (2000). Psychosocial antecedents of variation in girls’ pubertal timing: maternal depression, stepfather presence, and marital and family stress. Child Dev..

[28] Jean RT, Wilkinson A, v, Spitz MR, Prokhorov A, Bondy M, Forman MR. (2011). Psychosocial risk and correlates of early menarche in Mexican-American girls. Am J Epidemiol..

[29] Wronka I, Pawlińska-Chmara R (2005). Menarcheal age and socio-economic factors in Poland. Ann Hum Biol..

[30] Padez C (2003). Social background and age at menarche in Portuguese university students: A note on the secular changes in Portugal. Am J Hum Biol..

[31] Ghare Naz MS, Farahmand M, Dashti S, Tehrani FR (2022). Factors affecting menstrual cycle developmental trajectory in adolescents: A narrative review. Int J Endocrinol Metab..

[32] Papadimitriou A (2016). The evolution of the age at menarche from prehistorical to modern times. J Pediatr Adolesc Gynecol..

[33] Anikwe CC, Mamah JE, Okorochukwu BC, Nnadozie UU, Obarezi CH, Ekwedigwe KC. Age at menarche, menstrual characteristics, and its associated morbidities among secondary school students in Abakaliki, Southeast Nigeria. Heliyon. 2020;6(5) 10.1016/j.heliyon.2020.e04018 PMC7268279 32518847

[34] Dambhare DG, Wagh SV, Dudhe JY (2012). Age at menarche and menstrual cycle pattern among school adolescent girls in Central India. Glob J Health Sci..

[35] Kiran A, Geary RS, Gurol-Urganci I, Cromwell DA, Bansi-Matharu L, Shakespeare J (2018). Sociodemographic differences in symptom severity and duration among women referred to secondary care for menorrhagia in England and Wales: a cohort study from the National Heavy Menstrual Bleeding Audit. BMJ Open..

[36] Li H, Gibson EA, Jukic AMZ, Baird DD, Wilcox AJ, Curry CL (2023). Menstrual cycle length variation by demographic characteristics from the apple Women’s health study. NPJ Digit Med..

[37] Ohde S, Tokuda Y, Takahashi O (2008). Dysmenorrhea among Japanese women. Int J Gynecol Obstet..

[38] Nohara M, Momoeda M, Kubota T (2011). Menstrual cycle and menstrual pain problems and related risk factors among Japanese female workers. Ind Health..

[39] Haidari F, Akrami A, Sarhadi M (2011). Prevalence and severity of primary dysmenorrhea and its relation to anthropometric parameters. Tums-hayat..

[40] Patel V, Tanksale V, Sahasrabhojanee M (2006). The burden and determinants of dysmenorrhoea: a population-based survey of 2262 women in Goa, India. BJOG..

[41] Tavallaee M, Joffres MR, Corber SJ (2011). The prevalence of menstrual pain and associated risk factors among Iranian women. J Obstet Gynaecol Res..

[42] Kumari S, Sachdeva A. Patterns and predictors of premenstrual symptoms among females working in a psychiatry hospital. Scientifica. 2016:1–7. 10.1155/2016/6943852 PMC4884805 27293977

[43] AlQuaiz A, Albugami M, Kazi A, Alshobaili F, Habib F, Gold EB (2022). Dietary, psychological and lifestyle factors associated with premenstrual symptoms. Int J Women's Health..

[44] James AH (2016). Heavy menstrual bleeding: work-up and management. Hematology Am Soc Hematol Educ Program..

[45] Lee EJ, Ahn JE, Ryu JM, Jeong YY, Choi YS (2023). Association between patients’ self-judgement, coagulated menstrual blood, and menorrhagia: results from a questionnaire survey and blood test analysis. Medicina (Kaunas)..

[46] Nelsen LM, Lenderking WR, Pokrzywinski R, Balantac Z, Black L, Pokras S (2018). Experience of symptoms and disease impact in patients with adenomyosis. Patient..

[47] Wu S, Wang R, Zhao Y, Ma X, Wu M, Yan X (2013). The relationship between self-rated health and objective health status: a population-based study. BMC Public Health..

[48] Jylhä M (2009). What is self-rated health and why does it predict mortality? Towards a unified conceptual model. Soc Sci Med..

[49] Veenstra G (2011). Race, gender, class, and sexual orientation: intersecting axes of inequality and self-rated health in Canada. Int J Equity Health..

[50] Pedrós Barnils N, Eurenius E, Gustafsson PE (2020). Self-rated health inequalities in the intersection of gender, social class and regional development in Spain: exploring contributions of material and psychosocial factors. Int J Equity Health..

[51] Cislaghi B, Cislaghi C (2019). Self-rated health as a valid indicator for health-equity analyses: evidence from the Italian health interview survey. BMC Public Health..

[52] Teperi J, Rimpelä M (1989). Menstrual pain, health and behaviour in girls. Soc Sci Med..

[53] Ferguson KE (2017). Feminist theory today. Annu Rev Polit Sci..

[54] Bobel C. New blood: Third-wave feminism and the politics of menstruation [Internet]. New Blood: Third-wave Feminism and the Politics of Menstruation. Rutgers University Press. 2010.

[55] Magnay JL, O’Brien S, Gerlinger C, Seitz C (2018). A systematic review of methods to measure menstrual blood loss. BMC Womens Health..

[56] Marco Hernández M, Benítez R, Medranda I, Pizarro C, Méndez MJ (2008). Variaciones fisiológicas normales del desarrollo puberal: edad del inicio puberal, edad de la menarquia y talla. An Pediatr (Engl Ed)..

[57] De Sanctis V, Rigon F, Bernasconi S, Bianchin L, Bona G, Bozzola M (2019). Age at Menarche and Menstrual Abnormalities in Adolescence: Does it Matter? The Evidence from a Large Survey among Italian Secondary Schoolgirls. Indian J Pediatr..

[58] Parent AS, Teilmann G, Juul A, Skakkebaek NE, Toppari J, Bourguignon JP (2003). The timing of Normal puberty and the age limits of sexual precocity: variations around the world, secular trends, and changes after migration. Endocr Rev..

[59] Biro FM, Pajak A, Wolff MS, Pinney SM, Windham GC, Galvez MP (2018). Age of menarche in a longitudinal US cohort. J Pediatr Adolesc Gynecol..

[60] Wahab A, Wilopo SA, Hakimi M, Ismail D. Declining age at menarche in Indonesia: a systematic review and meta-analysis. Int J Adolesc Med Health. 2018;32(6). 10.1515/ijamh-2018-0021 30256760

[61] Marván ML, Catillo-López RL, Alcalá-Herrera V, del Callejo D (2016). The decreasing age at menarche in Mexico. J Pediatr Adolesc Gynecol..

[62] Sinai T, Bromberg M, Axelrod R, Shimony T, Stark AH, Keinan-Boker L (2020). Menarche at an earlier age: results from two National Surveys of Israeli youth, 2003 and 2016. J Pediatr Adolesc Gynecol..

[63] Lakshman R, Forouhi NG, Sharp SJ, Luben R, Bingham SA, Khaw KT (2009). Early age at menarche associated with cardiovascular disease and mortality. J Clin Endocrinol Metab..

[64] Goldberg M, D’Aloisio AA, O’Brien KM, Zhao S, Sandler DP (2020). Pubertal timing and breast cancer risk in the sister study cohort. Breast Cancer Res..

[65] Collaborative Group on Hormonal Factors in Breast Cancer (2012). Menarche, menopause, and breast cancer risk: individual participant meta-analysis, including 118 964 women with breast cancer from 117 epidemiological studies. Lancet Oncol..

[66] Piran N (2020). The menarche journey: embodied connections and disconnections. The Palgrave handbook of critical menstruation studies.

[67] Alcalá-Herrera V, MaL M (2014). Early menarche, depressive symptoms, and coping strategies. J Adolesc..

[68] Hapangama DK, Bulmer JN (2016). Pathophysiology of heavy menstrual bleeding. Womens Health (Lond Engl)..

[69] Gao Y, Hong X, Wang Z, Zhu Y (2018). Endometrial receptivity and conception outcome among women with light menstrual bleeding of unidentified etiology. Int J Gynaecol Obstet..

[70] Su S, Yang X, Su Q, Zhao Y. Prevalence and knowledge of heavy menstrual bleeding among gynecology outpatients by scanning a WeChat QR code. PLoS One. 2020;15(4). 10.1371/journal.pone.0229123 PMC7117654 32240178

[71] Fraser IS, Mansour D, Breymann C, Hoffman C, Mezzacasa A, Petraglia F (2015). Prevalence of heavy menstrual bleeding and experiences of affected women in a European patient survey. Int J Gynecol Obstet..

[72] Mansour D, Hofmann A, Gemzell-Danielsson K (2021). A review of clinical guidelines on the Management of Iron Deficiency and Iron-Deficiency Anemia in women with heavy menstrual bleeding. Adv Ther..

[73] Harvey LJ, Armah CN, Dainty JR, Foxall RJ, Lewis DJ, Langford NJ (2005). Impact of menstrual blood loss and diet on iron deficiency among women in the UK. Br J Nutr..

[74] Breymann C, Auerbach M (2017). Iron deficiency in gynecology and obstetrics: clinical implications and management. Hematology Am Soc Hematol Educ Program..

[75] Karlsson TS, Marions LB, Edlund MG (2014). Heavy menstrual bleeding significantly affects quality of life. Acta Obstet Gynecol Scand..

[76] Sumpter C, Torondel B (2013). A systematic review of the health and social effects of menstrual hygiene management. PLoS One..

[77] Elledge MF, Muralidharan A, Parker A, Ravndal KT, Siddiqui M, Toolaram AP (2018). Menstrual hygiene management and waste disposal in low and middle income countries—a review of the literature. Int J Environ Res Public Health..

[78] Sommer M, Hirsch JS, Nathanson C, Parker RG (2015). Comfortably, safely, and without shame: defining menstrual hygiene management as a public health issue. Am J Public Health..

[79] Armour M, Parry K, Manohar N, Holmes K, Ferfolja T, Curry C (2019). The prevalence and academic impact of dysmenorrhea in 21,573 Young women: A systematic review and Meta-analysis. J Women's Health..

[80] Weissman AM, Hartz AJ, Hansen MD (2004). The natural history of primary dysmenorrhoea: a longitudinal study. BJOG..

[81] Unsal A, Ayranci U, Tozun M (2010). Prevalence of dysmenorrhea and its effect on quality of life among a group of female university students. Ups J Med Sci..

[82] Sjöberg NO. Dysmenorrhea and uterine neurotransmitters. Acta Obstet Gynecol Scand. 1979; 87:57–9. 10.3109/00016347909157791 37691

[83] Sundell G, Milsom I, Andersch B (1990). Factors influencing the prevalence and severity of dysmenorrhoea in young women. Br J Obstet Gynaecol..

[84] Ramos-Pichardo JD, Ortega-Galán ÁM, Iglesias-López MT, Abreu-Sánchez A, Fernández-Martínez E (2020). Why do some Spanish nursing students with menstrual pain fail to consult healthcare professionals?. Int J Environ Res Public Health..

[85] Abreu-Sánchez A, Ruiz-Castillo J, Onieva-Zafra M, Parra-Fernández M, Fernández-Martínez E (2020). Interference and impact of dysmenorrhea on the life of Spanish nursing students. Int J Environ Res Public Health..

[86] Armour M, Smith CA, Steel KA, Macmillan F (2019). The effectiveness of self-care and lifestyle interventions in primary dysmenorrhea: a systematic review and meta-analysis. BMC Complement Altern Med..

[87] Valls LC (2021). Mujeres invisibles para la medicina.

[88] McMillan C, Jenkins A (2016). “A magical little pill that will relieve you of your womanly issues”: what young women say about menstrual suppression. Int J Qual Stud Health Well-being..

[89] Hasson KA (2020). Not a “real” period?: social and material constructions of menstruation. The Palgrave handbook of critical menstruation studies.

[90] Wood JM (2020). (in)visible bleeding: the menstrual concealment imperative. The Palgrave handbook of critical menstruation Studies.

[91] Sommer M, Lee C, Liu D, Gruer C (2020). The extent to which menstruation-related issues are included in graduate-level Public health curricula. Front Public Health.

[92] Lete I, Dueñas JL, Serrano I, Doval JL, Martínez-Salmeán J, Coll C, Pérez-Campos E, Arbat A (2011). Attitudes of Spanish women toward premenstrual symptoms, premenstrual syndrome and premenstrual dysphoric disorder: results of a nationwide survey. Eur J Obstet Gynecol Reprod Biol.

[93] Johnston-Robledo I, Chrisler JC (2020). The menstrual mark: menstruation as social stigma. The Palgrave handbook of critical menstruation studies.

[94] Ussher J (2006). Managing the Monstruous feminine: regulating the reproductive body, women and psychology.

[95] King S (2020). Premenstrual syndrome (PMS) and the myth of the irrational female. The Palgrave handbook of critical menstruation studies.

[96] Ussher J (2011). The madness of women.

[97] Lahiri-Dutt K (2015). Medicalising menstruation: a feminist critique of the political economy of menstrual hygiene management in South Asia. Gender, Place and Culture [Internet].

[98] Young K, Fisher J, Kirkman M (2015). Women’s experiences of endometriosis: A systematic review and synthesis of qualitative research. J Family Plan Reprod Health Care..

[99] Evans J, Ziebland S, McPherson A (2006). Minimizing delays in ovarian cancer diagnosis: an expansion of Andersen’s model of “total patient delay.”. Fam Pract..

[100] Crenshaw K (1989). Demarginalizing the intersection of race and sex: A Black feminist critique of antidiscrimination doctrine, feminist theory and antiracist politics.

[101] Lentz G, Lobo R, Gershenson D (2012). Comprehensive gynecology.

[102] Taylor HS, Pal L, Seli E (2020). Speroff’s clinical gynecologic endocrinology and infertility.

[103] Kwak Y, Kim Y, Baek KA (2019). Prevalence of irregular menstruation according to socioeconomic status: A population-based nationwide cross-sectional study. PLoS One..

[104] Medina-Perucha L, López-Jiménez T, Holst AS, Jacques-Aviñó C, Munrós-Feliu J, Martínez-Bueno C (2022). Self-reported menstrual alterations during the COVID-19 Syndemic in Spain: A cross-sectional study. Int J Women's Health..

[105] Figà-Talamanca I (2006). Occupational risk factors and reproductive health of women. Occup Med (Lond)..

[106] García Calvente M, del Río LM, Marcos Marcos J (2011). Desigualdades de género en el deterioro de la salud como consecuencia del cuidado informal en España. Gac Sanit..

[107] García-Calvente M, Mateo-Rodríguez I, Maroto-Navarro G (2014). El impacto de cuidar en salud y la calidad de vida de las mujeres. Gac Sanit..

