# Supplementary material for: Menstrual characteristics and associations with sociodemographic factors and self-rated health in Spain: a cross-sectional study
Source: BMC Womens Health. 2024 Feb 3;24:88. doi: 10.1186/s12905-023-02840-z (PMC10838449; doi:10.1186/s12905-023-02840-z)
Supplement: Supplementary file 1 — Additional file 1. [file 12905_2023_2840_MOESM1_ESM.docx]

**Supplementary File 1. Equity and Menstrual Health Survey**

**Sociodemographic questions**

1. Age: __________
2. Country of birth: ___________
3. What is your administrative situation in Spain?
   1. Spanish Nationality
   2. Permanent residency
   3. Temporal residency
   4. No permit or permit in process
4. What is your employment situation? *You may click more than one option*
   1. Work full-time
   2. Work part-time
   3. Self-employed
   4. Study full-time
   5. Study part-time
   6. Unemployment/COVID19 benefits
   7. Housework or caring for others (not getting paid)
   8. Pension (including retirement)
5. Have you ever had financial difficulties in the last year?
   1. Yes, always
   2. Yes, many times
   3. Yes, some times
   4. Yes, a few times
   5. No, never
6. Do you care for other people (e.g., minors)?
   1. Yes
   2. No
7. What studies have you finalized?
   1. Primary education
   2. Secondary education
   3. Basic professional training
   4. Middle professional training
   5. Superior professional training
   6. University studies
   7. Postgraduate studies
   8. No studies finalized
8. What gender do you identify with?
   1. Woman
   2. Man
   3. Non-binary
   4. Other
9. Do you identify as a trans person?
   1. Yes
   2. No
   3. I do not know

**About your menstruations**

1. How old were you when you first menstruated? _______
2. How are your menstruations usually? *You may click more than one option*
   1. Bleeding less than 25ml (in total each menstruation)
      (≤6 regular tampons or pads, or less than 1 full 20ml menstrual cup)
   2. Bleeding between 25-80ml (in total each menstruation)
      (7-15 regular tampons or 7-19 regular pads, or between 1 and 4 full 20ml menstrual cups)
   3. Bleeding more than 80ml (in total each menstruation)
      (≥ 16 regular tampons or ≥ 20 regular pads, or more than 4 full 20ml menstrual cups)
   4. I have menstrual clots (non-liquid)
3. How long do your menstruations usually last?
   1. Less than 2 days
   2. Between 2 and 7 days
   3. More than 7 days
4. How often do you usually menstruate?
   1. Every 21 days or less
   2. Every 21-35 days
   3. Every 35 days or more
5. Do you experience changes in mood (e.g., sadness or irritability) and/or physically (e.g., tiredness or liquid retention) 1 or 2 weeks before menstruating?
   1. Yes, always
   2. Yes, many times
   3. Yes, some times
   4. Yes, a few times
   5. No, never
6. Usually, how intense is your menstrual pain, in a scale between 1 (not painful) and 10 (very painful)?

| 1 | 2 | 3 | 4 | 5 | 6 | 7 | 8 | 9 | 10 |
| --- | --- | --- | --- | --- | --- | --- | --- | --- | --- |

1. What do you do when you have menstrual pain? *You may click more than one option*
   1. Use of analgesics (e.g., ibuprofen or paracetamol)
   2. Use of hormonal contraception for menstrual pain
   3. Use of natural remedies (e.g., infusions or thermic pillow)
   4. Cannot afford menstrual pain management products
   5. I do not do anything
   6. I do something else: __________
   7. I do not have menstrual pain
   8. I do not know what to do
2. Have you ever been diagnosed of any of the following conditions? *You may click more than one option*
   1. Anemia
   2. Iron deficiency
   3. Uterine myomas
   4. Endometrial polyps
   5. Endometriosis/adenomyosis
   6. Polycystic ovary syndrome
   7. Premenstrual syndrome/dysphoric premenstrual disorder
   8. Ovarian or fallopian tube cancer
   9. Uterine cancer
   10. Cervical cancer
   11. Breast cancer
3. No diagnosis
4. At this time, have you stopped menstruating for more than 1 month (including due to health conditions or pregnancy/breastfeeding)?
   1. Yes
   2. No
5. Have you ever taken hormonal contraceptives?
   1. Yes, I take them currently
   2. Yes, I have taken them in the past
   3. No, never
6. How would you rate your general health status?
   1. Excellent
   2. Very good
   3. Good
   4. Fair
   5. Poor
